# Medical decision-making experiences of persons with dementia and their carepartners: a qualitative study

**DOI:** 10.1186/s12904-025-01710-9

**Published:** 2025-04-09

**Authors:** Thalia Porteny, Mary Lynch, Audrey Covaleski, Jennifer Tjia, Priscilla Gazarian, Amanda J Reich, Stephen Perez, Kristen Kennefick, Joel S. Weissman, Keren Ladin

**Affiliations:** 1 Department of Health Policy and Management, Mailman School of Public Health, Columbia University, 722 West 168th St, New York, NY 10035 USA; 2 Research on Ethics, Aging, and Health (REACH Lab), Tufts University, Medford, MA USA; 3 UMass Boston, Boston, MA USA; 4 UMass Chan Medical School, Worcester, MA USA; 5 Center for Surgery and Public Health, Brigham and Women’s Hospital, Boston, MA USA; 6 Harvard Medical School, Boston, MA USA; 7 Department of Community Health, Tufts University, Medford, MA USA

**Keywords:** ADRD, Dyads, Decisional quality, ACP, PWD

## Abstract

**Background:**

Persons with dementia (PWD) and their carepartners must often make complex medical decisions, weighing the benefits of medical (surgical and non-surgical) interventions with uncertainty regarding outcomes, both dementia- and non-dementia related, in the short-term and long-term. This study informs gaps in clinical guidance for patient-centered decision-making about medical and surgical interventions for PWD and advancecare planning.

**Methods:**

We conducted a qualitative study using thematic analysis based on semi-structured interviews with PWD and carepartners.

**Results:**

We interviewed 30 participants (9 PWD, 21 carepartners). Four themes were identified (with related subthemes): 1) PWD and carepartners varied in using decision-making approaches for medical interventions for PWD (a) variations in views about decision-making load; (b) Progressive involvement of carepartners in ACP decision-making as cognition erodes; 2) medical intervention decisions were an inflection point to evaluate values for dyads and involved tradeoffs with implications for end-of-life care and quality of life 3) lack of discussion with clinical team about impact of medical interventions on dementia burdened dyads; 4) decisional quality was facilitated by: (a) a trusting relationship with clinicians; and (b) a multidisciplinary team approach.

**Conclusion:**

Most patients with mild-to-moderate dementia and carepartners approach medical intervention decision-making guided by their understanding of the dementia prognosis, but the risks of medical interventions are often unaddressed in discussions with the clinical team, sometimes burdening dyads with undesirable consequences to their quality-of-life. Clinicians should provide dementia-related risks regarding medical intervention outcomes to best facilitate decision-making conversations and advance care planning.

**Supplementary Information:**

The online version contains supplementary material available at 10.1186/s12904-025-01710-9.

## Introduction

Dementia is a set of progressive, neurodegenerative conditions affecting over 6.5 million Americans, and its incidence is expected to grow [1]. Among older persons with dementia (PWD), many non-surgical and surgical medical interventions pose a heightened but underappreciated risk for cognitive decline and mortality compared with older adults without dementia [2, 3]. Commonly recommended non-surgical medical interventions among older adults, such as screening tests for cancer (e.g. colonoscopies) and others, have greater risk of harm in patients with PWD than those without, including distress from the tests and complications from follow-up testing and treatment [4]. PWD are also at increased risk of adverse post-surgical outcomes, including delirium, sedation impairment, post-operative infection, prolonged recovery time, and hospital readmission [5, 6]. For example, one study of 626 persons undergoing hip fracture repair report a one-year post-surgical mortality of 33.8% for PWD, compared to 18.7% for those without dementia [7]. In this context, PWD and their carepartners must weigh both dementia- and non-dementia related outcomes, including overall health in the short-term and long-term, when consenting to interventions.

Limited guidance exists for clinicians regarding how to best describe the risks and benefits of proposed medical interventions with older PWD and their carepartners [8, 9]. To date, the literature mostly focuses on promoting advance care planning (ACP). ACP is a process designed to help patients and their carepartners discuss their preferences for their current and future medical care based on their values and goals [10], in order to help preserve patient autonomy by having formal, preference-clarifying conversations between patients, carepartners, and clinicians. Despite high complications rates for PWD undergoing medical interventions, ACP is seldom completed as part of discussions with PWD [11, 12].

There is little insight into how PWD and carepartners can be better supported in medical intervention decision making and ACP from their perspective. Yet, PWD often express a strong desire to participate in decision-making regarding medical interventions, such as for cancer treatment [8] and Alzheimer’s disease treatment [13]. PWD also report a preference for being involved in end-of-life care discussed in ACP, such as a DNR order [14, 15]. The limited studies on the carepartners’ perspective elucidate that some prefer for medical procedures, such as cancer screenings, to stop as dementia progresses, and are open to discussions of screening cessation that focus on quality of life, burdens and benefits, but these rarely happen [4] and carepartners for PWD are often left feeling excluded from medical intervention decision-making [16, 17]. Still, little is known about how PWD and carepartners approach these decisions together.

Owing to inevitable cognitive decline for PWD, there are differing levels of PWD and carepartner decision-making involvement, reflective of their cognitive ability and relationship dynamics [18]. The range includes ‘supported decision-making’, which refers to a person living with dementia (PWD)-led process by which PWD make their own decisions with the support from a trusted other [19]. The trusted other, often being the carepartner, work to integrate the patient as much as possible despite a PWD’s impaired decision-making capacity, as well as ‘surrogate decision-making’ which occurs when carepartners must make decisions based on their best knowledge of a PWD’s goals and values because of complete decisional impairment [15, 18, 20]. But less is known about how PWD and carepartners approach complex medical decision-making involvement as shifts occur due to disease progression. Concordantly, as shifts in decision-making dynamics take place, the effect of complex decision-making dynamics, disagreement, and discordant values among PWD and their carepartner on ACP *and treatment decisions* remain incompletely understood [21, 22].

Current studies acknowledge the need to develop clinician ACP trainings and further research on medical intervention decision-making for PWD [21, 23, 24]. This study informs gaps in clinical guidance for patient-centered decision-making about high-risk medical (surgical and non-surgical) interventions for PWD from PWD and the carepartner perspective.

## Methods

We conducted a qualitative study using thematic analysis based on semi-structured interviews with PWD and carepartners.

### Participant recruitment

PWD and carepartners were recruited via convenience sampling from three health systems in the Northeast and Mountain regions of the United States. Inclusion criteria for patients included being a Medicare beneficiary (e.g. US federal health insurance for people 65 and older), English speaking, and having a documented diagnosis of mild-to-moderate dementia as determined by their neurologist. Patients were identified by providers through the participating sites. Carepartners were identified by patient participants as non-clinician individuals with whom they are most likely to discuss medical decisions. Only one carepartner was included for each person living with dementia and no people with dementia participated without a carepartner. Some carepartners who cared for a PWD who was either unwilling to be interviewed or did not pass the cognitive screener chose to still participate in the study. Purposive sampling criteria included recruiting patients with early dementia that passed the cognitive screening.

Recruitment strategies at each site reflected the preferences of the health system and all included an opt-in/opt out letter, followed by a phone call. At one site, the health system’s NIA-designated Alzheimer’s Disease Research Center was used to identify patients from a longitudinal cohort study based having a Clinical Dementia Rating Score (0 normal to 2 moderate dementia) of 0.5-2. At another site, the local study champion selected eligible patients from site’s dementia program for patients and carepartners. At the final site, patients were recruited from the site’s geriatrics center and Alzheimer’s disease center. Eligible geriatric center patients were identified in the system’s electronic medical record. Patient contact was provided to the study team, and an opt-in/opt-out letter was sent, followed by a phone call.

PWD participants were screened for research capacity using an 8-item cognitive assessment designed to evaluate their understanding of the study’s purpose, consent procedures, and the voluntary nature of participation. To be eligible to provide consent, PWD participants were required to achieve a full score (8/8, 100%). This screening tool was developed in accordance with local IRB requirements (See supplementary material). Following an approach used in prior research [25], providers and health systems assisted in identifying individuals with mild to moderate dementia as potential participants. Carepartners were eligible to participate whether or not their associated PWD was eligible based on the cognitive assessment. This study was approved by the Partners HealthCare Institutional Review Board.

### Data collection

The interview guide was pilot tested and iteratively revised. Data collection occurred between April 2019 and March 2021. Each interview was completed by phone by either a researcher clinician with expertise in dementia or social science researchers who also had expertise in dementia (J.T., P.K.G., A.J.R., S.P.). Most of the interviews were carried out by two researchers. Participants provided verbal consent and completed a demographic survey. The interview lasted 38 min on average with a standard deviation of 12.63 min.

A semi-structured interview guide was created by J.T., P.K.G., A.J.R., S.P. KL, a multidisciplinary team comprised of social scientists with expertise in qualitative methods and clinicians with expertise in geriatric palliative care and dementia care (see Supplementary Material). Open-ended questions examined how patients and carepartners engaged in medical decision-making, as well as what goals, values and preferences were prioritized in making these decisions. Participants were also asked to choose on a scale of 1 to 10, what number would characterize their level of shared decision-making and what circumstances would shift their decision-making load now or in the future. Other questions included descriptions of ACP conversations.

### Analysis

We adopted a post-positivist framework, which focuses on maintaining objectivity, reliability, and accuracy in coding while minimizing researcher bias. This approach involves strategies such as using structured codebooks, having multiple coders independently analyzing the same data, calculating intercoder agreement, and applying consensus coding methods [26].

Interviews were audio-recorded and professionally transcribed verbatim, then uploaded to NVivo 11 (QSR International; Melbourne, Australia). Sampling of participants continued until thematic saturation was achieved and confirmed through deliberation by the research team [27]. The preliminary codebook was concept driven, based on interview questions and revised over time. Codes were deductively pre-defined based on the interview guide as well as inductively allowing for emergent codes [27]. T.P., A.C., and M.L independently coded 6 of 30 interviews (20%) line-by-line and iteratively revised the codebook to include emergent codes. Codes were then amended and organized into categories through a consensus process to reflect the range and variability of subthemes, and to characterize both confirmatory and contradictory narratives [28, 29]. This study follows the Consolidated Criteria for Reporting Qualitative Health Research (Supplementary material) [30].

## Results

Overall, 30 participants completed interviews: 9 patients with mild-to-moderate ADRD and 21 carepartners. Of the carepartners, 5 (23.8%) were children of the patients and 16 (76.2%) were spouses/partners. All the patients’ carepartners were interviewed (total of 9 dyads). Sample characteristics can be found in Table 1.

**Table 1:** Participant characteristics

Participant Characteristics (*n* = 30)	Patients (*n* = 9)	Carepartners (*n* = 21)
Age, mean (SD)	74.0 (9.6)	69.9 (10.4)
45–54	0 (0.0%)	1 (4.8%)
55–64	2 (22.2%)	8 (38.1%)
65–74	1 (11.1%)	3 (14.3%)
75–84	6 (66.7%)	9 (42.8%)
No Reply	0 (0.0%)	1 (4.8%)
Gender, N (%)		
Female	0 (0.0%)	19 (90.0%)
Male	9 (100.0%)	1 (4.8%)
No Reply	0 (0.0%)	1 (4.8%)
Race, N (%)		
White, non-Hispanic	9 (100.0%)	17 (81.5%)
White, Hispanic	0 (0.0%)	1 (4.8%)
Asian	0 (0.0%)	0 (0.0%)
Black or African American	0 (0.0%)	2 (9.5%)
Mixed	0 (0.0%)	0 (0.0%)
No reply	0 (0.0%)	1 (4.8%)
Ethnicity, N (%)		
Hispanic	0 (0.0%)	1 (4.8%)
Non-Hispanic	9 (100.0%)	19 (90.0%)
No Reply	0 (0.0%)	1 (4.8%)
Education Level, N (%)		
High School	2 (22.2%)	3 (14.3%)
< High School	7 (78.8%)	18 (86.7%)
Child of participant (%)		5 (23.8%)
Spouse/partner of participant (%)		16 (76.2%)

We found four overarching themes (with related subthemes) characterizing decision-making dynamics around medical interventions for older PWD and their carepartners: 1) PWD and carepartners varied in using decision-making approaches for medical interventions for PWD (a) divergent and convergent views about distribution of decision-making load; (b) accommodating shifts from shared, to supported, to surrogate ACP decision-making; 2) medical intervention decisions were an inflection point to evaluate values for dyads and involved tradeoffs with implications for end-of-life care and quality of life 3) lack of discussion with clinical team about impact of medical interventions on dementia burdened dyads; 4) decisional quality were facilitated by: (a) a trusting relationship with clinicians; and (b) a multidisciplinary team approach. (Table 2)

**Table 2:** Notable quotes from patients and carepartners

**PWD and carepartners vary in decision-making approaches for interventions for PWD**
“We talk all the time about our feelings, and she pretty much leaves the finances and that kind of thing up to me, and that’s fine with her. I mean, she trusts me, and she knows that I’m going to– one of our main goals was to have a good amount of money that we could pass on to our son.” (CP #6).
“I’m making all the–[decisions]… I would be making all the decisions for (PT #8) and I, most likely” (CP #20) “Well, he’s still involved in the decision-making. I still give him choices, I guess, currently. But they could change in the future, and he verbally will say to me, “(CP #20), I don’t know what you’re talking about,” or, “Could you just decide for me?” Whether it’s for a text you received or to call someone back, if he’s getting agitated or frustrated, then I would be making the decision, I guess, for him…But he’s very much a part of the process, and he does let me know if he just doesn’t understand” (CP #20) “(CP #20) does all of it. I mean, that’s what happens every day” (PT #8)
**Lack of discussion about impact of medical intervention on dementia posed a barrier to decision-making and burdened dyads**
“I’ve asked them, all the reading that I’ve done, what stage is he in, and the doctor at [institution] said, “Don’t even look at stages,” he said, “we don’t think of it that way.” No, we haven’t gotten a lot of answers about anything…Well, I mean how quickly is this going to progress? They don’t know. How long is he going to be able to function as well as he is now? They don’t know. I mean I don’t ever get answers to anything.”(CP #13)
“I wish [he] would have told me a little bit more about how the general anesthesia and the hospital experience would have made her. Because she was really agitated. She was really confused.” (CP #7)
“Oh, no. No. No. And we have a doctor at [institution] who is aware. We go like twice a year to see him, but anytime we have questions or something. But that never came under consideration at all, no. I didn’t believe that the surgeries -- I never even asked anybody. I didn’t believe that the surgeries should be put off, and nor did he, and nor did the doctors (CP #11)
“The main takeaway is that you really have to advocate on behalf of your loved ones and yourself in terms of medical care and whatnot. I mean, you have to ask a lot of questions. In some cases, sometimes you have to challenge things.” (CP #9)
**Medical intervention decisions were an inflection point to evaluate values for dyads and involved tradeoffs with implications for end-of-life care and quality of life**
“So I can definitely imagine a situation where if [the patient] were really sick, and let’s say she had a real bad stomach ailment or something like that, and she just didn’t want to have any medical care, see any care at all, I can imagine that would be a situation where I would override what she wishes in that moment and seek care.” (CP #9)
“And you get to a certain age, it’s like so you can have a lot of chemo and get yourself all together and then die tomorrow because of your age. So that’s a factor in decisions with regard to surgery. If you can live with stuff– I think both [the patient] and I are of the same mind. If you can live with it, then do so.” (CP #1)
**Decisional quality were facilitated by a trusting relationship with clinicians and a multidisciplinary team approach.**
Trusting relationship with clinicians
“Yeah, just work with your medical team. That’s the best advice I have, yeah. They’ve been the biggest help to me, just knowing they’re there; I can call and talk to people, and… good help that way” (CP #4).
“Some do. Some don’t. I think the best do understand that. And I think those who don’t are just sort of blindly– you become a series of body parts that needs to be fixed, and then that’s how it’s approached. But I think, really, superior physicians really get it about the quality of life. And it’s a real factor, I think, when you’re making those kinds of decisions” (CP #1).
Multidisciplinary Team Approach
“And then we would break off into separate groups. And all the caregivers would go into one room, and the patients, I guess, whatever, our loved ones, would go in another room. And I always found it so helpful, because in that support group of just the caregivers getting together, or through Surrey, I guess, also. There was a caregiver support group there also. That, to me, would be the place to have those discussions” (CP #1).
“That they have a nurse advocate for the patient so that when these doctors come in you have someone who is listening and documenting what is going on. That’s very important” (CP #2).

### PWD and carepartners vary in using decision-making approaches for medical interventions for PWD

#### Variation in views about distribution of decision-making load

Participants described a spectrum of approaches to integrating carepartners into decision-making around medical interventions for persons with mild and moderate dementia: ACP-informed shared decision making, supported decision making and surrogate decision making. Some Dyads expressed different perspectives about how the decision-making load was distributed between patient and carepartner regarding medical interventions. For example, when a carepartner was prompted to rate on a scale of 1 to 10, with 1 meaning the patient makes all of his healthcare decisions alone, and 10 meaning carepartner makes all decisions, the carepartner said *“Probably at least I do 8.”* Her husband a PWD, had a different perspective. He rated decision making as *“5*,* or maybe 6*,* a little bit more to my favor.”* (PT #2 and CP#5).

Other participants, however, expressed similar views on how medical intervention decisions are made. One carepartner explained that shared values and her trusting spousal relationship enabled possible future surrogate decision making, whereby the carepartner would approximate an autonomous decision. *“We’ve been married 57 years and probably know each other as well as you could know each other. We’re both very conservative. We both like each other*,* luckily. So I feel like I would do for him what I would do for me.” (CP #8)* Her husband echoed this mutual trust, believing that she would adhere to their shared values and operate with their best interest in mind, if he could no longer advocate for himself: *“I think if I were in a position where I couldn’t make decisions*,* I’d feel like [she] could do any of it. She could take it over completely.” (PT# 3).*

#### Progressive involvement of carepartners in ACP decision-making as cognition erodes

Participants described a progressive involvement of carepartners in ACP decision-making as dementia progressed. In describing her husband’s shift towards needing supportive decision-making around ACP, one carepartner explained: *“He was having a hard time putting all of the information together*,* so I kind of stepped in to help him make decisions*,* which I had never done before. He always made all those decisions on his own…” (CP #10).* Participants emphasized the need of early ACP conversations for successful shifts to supported decision-making, which allowed the patient to play an active role in designating their health care proxy, and expressing their ACP preferences. When probed about the timing of ACP conversations, one carepartner explained: *“… we’ve had everything in place now for probably close to two years… proactively done at her suggestion” (CP #9).*

Some carepartners in the more supported decision-making role aimed to build a balanced dynamic with their PWD by frequently encouraging the patient to engage in ACP conversations, prompting them to verbalize their values and goals. A carepartner explained:*“I would say I involve [my husband] in the process as much as possible. I’ve never wanted to take away his independence --making those kinds of decisions without him*,* until the time comes that that’s not an option anymore” (CP #3).* Another carepartner explained that clear communication and having her spouse, a person with dementia, take an active role in making decisions around an intervention, was empowering for him. *“It’s very important to him. He needs to feel that he’s part of the decision…. he’s very much a part of the process*,* and he does let me know if he just doesn’t understand*,*” (CP #20)*.

### Medical intervention decisions were an inflection point to evaluate values for dyads and involved tradeoffs with implications for end-of-life care and quality-of-life

Despite high risk of complications for PWD, few patients and carepartners engaged in formal goals of care or ACP discussions prior to their medical intervention decision-making process. When prompted about what values, goals, and preferences were most important during medical intervention decision-making for PWD, most participants framed decisions around the value of preserving the patient’s independence and quality-of-life as much as possible, rather than pointedly referring to cognitive function or preventing further cognitive decline. One caregiver stated, *“But what kind of quality of life is he going to have after that? Is he going to be worse off to where he’s never going to really come back at all mentally or physically or anything?” (CP14)* Another carepartner noted that her mother just *“wants to feel independent.” (CP #7)* Several patients described strong family values. What was most important to them as they approached medical intervention decisions was *“Keeping [themselves] healthy and [their] family happy. Not being a super burden on them.”* (PT #2) For many, medical intervention decision-making and potential tradeoffs posed an opportunity to evaluate their values which could help facilitate discussion of larger goals and preferences pertaining to future medical care. *“I think it’s important to start conversations about death and dying. And I mean*,* because that’s the context*,* I think*,* you talk about the decisions that are made with regard to surgery or even*,* I mean*,* any medical procedure.”* (CP #1).

Some dyads pursued medical interventions when it was intended to treat a major medical issue or significantly improve quality-of-life, despite the risk of complications associated with dementia. One carepartner explained that making the decision for her husband to undergo cataracts surgery: *“He’s almost legally blind without glass correction refraction. For us*,* it was an easy decision because he would be able to go in*,* and they could do the prescriptive implant*,* and he can see great*,* now.” (CP #7).* One carepartner explained that, when making decisions for her elderly mother: *“I think it’s a quality-of-life issue so that if the surgery is risky and if it would perhaps diminish the quality-of-life for a substantial period of time*,* I think we’d say no to it… She’s 81… because if you got 10 years left*,* you don’t want to spend 9 of it recovering from something*,*” (CP #1).*

Another dyad discussed the possibility of having an invasive medical intervention done to alleviate some symptoms, and determined that the procedure was too invasive and not worth the risk: *“[The patient] just felt uncomfortable with (the surgery). Ultimately*,* it was his decision. And we also found out that it wasn’t absolutely necessary*,*” (CP #3).* Other carepartners heavily weigh the risk of worsening cognitive function against the immediate health benefits of medical interventions: *“our main thing was*,* ‘how is this going to affect him cognitively” (CP #14).*

### Lack of discussion about impact of medical intervention on dementia posed a barrier to decision-making and burdened dyads

Medical intervention discussions focused on non-dementia outcomes, with little consideration for dementia-related outcomes. For many dyads, lack of discussion about the unpredictability of dementia prognoses and the impact of medical interventions on dementia posed a barrier to decision-making. Patients described receiving information about some aspects of recovery such as pain management, wound care, and quality-of-life, but not their primary concern of cognitive-related outcomes.

Dyads described that lack of discussion of cognitive decline following medical interventions was an unaddressed concern which impeded their ability to effectively consider the risks and benefits. One carepartner explained: *“I feel that the back surgery… he did not get all of the care that he needed for his situation. I must have told them every day. I said*,* ‘He has a memory problem…’ So*,* it was a bad experience*,* but he did have memory problems. And afterwards*,* they were worse.” (CP #2).* Another carepartner described the burden of developing a post-surgical recovery plan due to decision-making guidance for PWD not being dementia friendly. “*There’s not enough information on how difficult things are going to be post-surgically*,* what the advantages versus the disadvantages are with dementia patients*,* and how to make those decisions.” (CP #7).*

Participants described the emotional toll postoperative cognitive decline took on both the patient and family, especially when the risk of complications had not been explained. One carepartner explained her mother’s worsened cognition, after what she thought was a minor surgery: “*It was a type of surgery that we were kind of reassured it’s basically a minor surgery*,* I guess I don’t think anything’s always minor*,* -- it was difficult when she was sedated. It was terrible after she got out*,* and I think it was exacerbated by her dementia. She was really*,* really messed up after she got out of the sedation.” (CP #15)*.

Dyads also faced challenges owed to the difficulty of establishing a dementia prognosis such as medical risks or the best time to bring in a professional care-team for assistance with daily activities. *“I think what would be helpful would be if we knew where we were going and how soon we’re going to get there. And those are questions that no one can answer. The difficult part is there’s so many unknowns.” (CP #13).*

Some carepartners failed to understand the potential for a surgical-related cognitive decline: *“I would imagine that most of the surgeries*,* other than something like if you had brain surgery*,* it wouldn’t matter whether you had dementia or not. I don’t know for sure.” (CP #1).* Although there was uncertainty regarding the impact of a medical intervention on her father’s cognition, this carepartner appreciated when clinicians candidly described potential risks: “*We did know [cognition-related post-operative] risks. It was all explained to us that coming out of anesthesia*,* he might not have the same level of cognitive-ness as he did beforehand. Doctor was always very well in explaining all of that.” (CP #14).*

### Decisional quality was facilitated by a trusting relationship with clinicians and a multidisciplinary team approach

#### Trusting relationship with clinicians

Despite the uncertainty and obstacles dyads faced during decision-making, many stated that having a positive relationship with their clinician that involved trust, good communication, transparency, as well as access to supportive and informative resources increased their confidence in decision making. One participant said: “*If I had not had a doctor who listened and offered help*,* not only medically but how to handle all of these [dementia related] changes that were coming so quickly*,* I would not have made it*” (CP #10).

One person living with dementia explained that he depended on his clinician to provide accessible PWD resources and information to not only him but his family: “*I would want the team to be absolutely honest and explain what was going on to the family in as easy a way as possible… I certainly want them to be given a thorough explanation.*” (PT #9).

Additionally, patients and carepartners expressed appreciation for clinicians who initiated conversations about their overall well-being that went beyond diagnosis and prognosis, and helped to develop confidence in their clinician’s ability to understand their values and preferences for future medical care and end-of-life care. One carepartner said: *“[the patient is] very happy that she has somebody who gives more than 20 minutes*,* as most doctors do in their visits. She gets 40 minutes*,* and they can talk*,* and talk*,* and talk. So she enjoys that. She feels in good hands.” (CP #19).*

Conversely, patients and their carepartners noticed when clinicians were less involved, and generally expressed more dissatisfaction with their medical care. A carepartner shed light on feelings of discontent with her husband’s care, hoping that clinicians could provide more support during visits: *“I felt there was a sense of*,* and somebody told me this*,* ‘Diagnose and adios.’ He’s been diagnosed with the disease*,* yeah*,* go have a nice life.”(CP#3).*

#### Multidisciplinary team approach

Carepartners and patients found it helpful when clinicians connected them with other ADRD resources, such as support groups and social workers. Carepartners especially appreciated the opportunity to discuss their experience with others who could relate to their situation, provide support and offer advice. For instance, a carepartner expressed how instrumental her support group has been throughout her husband’s dementia progression, saying: *“Everybody who’s in the support group…has a spouse who has dementia…So it’s a matter of sharing so that we can anticipate what might be happening or to find out what is happening is fairly normal.… it’s helpful” (CP #5).*

Additionally, carepartners valued the interdisciplinary approach to dementia care because it provided well-rounded support for decision-making and, most importantly, a professional they could talk to about their experience. A participant in this study was a part of a multidisciplinary ADRD team at their hospital, which was a comfort throughout her husband’s dementia diagnosis. *“That group has just been so important in my life… the group consists of a pharmacist*,* a social worker - but she calls me every month; she’s probably the coordinator - and [the doctor]. There are a lot of resources available to me” (CP #10).*

## Discussion

This qualitative study characterizing decision-making experiences of persons with mild-to-moderate dementia and their carepartners found that PWD and carepartners want to know more about cognition-related declines when weighing the risks of non-dementia related medical interventions to quality-of-life improvements. Many PWD preferred to be actively engaged in decision-making, but carepartners were left having to undertake a more active role in ACP, if not done early, as their loved ones’ disease progressed. Moreover, some dyads reported that the uncertainty regarding dementia- and non-dementia related outcomes of procedures limited their ability to make informed decisions. Many dyads stated that they did not receive sufficient and timely information on the cognitive implications of surgical procedures during their decision-making process, despite the high value placed on this information, leaving them unprepared and burdened by dementia related outcomes. This gap underscores the need for clinical guidance on addressing dementia-related cognitive risks in surgical decision-making conversations, preferably from a trusted clinician.

Our findings are consistent with previous research which reports that clinicians often do not provide tailored information for PWD undergoing surgery, such as outlining the potential risks of worsening cognitive decline [31]. Moreover, clinicians report rarely completing preoperative screenings for frailty or dementia, as recommended by the American Society of Anesthesiologists, which could help indicate the degree of risk and guide discussions with patients [31]. This is not due to lack of knowledge on cognitive outcomes for PWD after surgery– in fact, several studies have noted the increased risk of poor outcomes and cognitive decline after surgery for PWD [32] and that this decline is accompanied by a significant emotional toll for both the patient and their loved ones [33]. Yet, although clinicians understand the importance of tailored discussions, they do not initiate them due to lack of training, and hospital programming [31, 34, 35]. At the same time, studies show that for PWD and their carepartners to be engaged in tailored discussions, they must have adequate health literacy so they can understand and act on the provided health information [36]. Our work can help inform clinicians on how to best educate and involve carepartners in the discussion of medical implications, when the cognitive decline of the PWD has progressed past the ability to make decisions, as well as people with dementia themselves [37, 38].

One of the key findings of our study demonstrates that decision-making around medical interventions is an inflection point for values discussions where dyads can evaluate their goals of care. But research indicates that this evaluation of values and ACP conversations need to happen earlier for PWD, ideally before significant disease progression and as an ongoing process to accurately inform medical decisions based on PWD’s wishes [39]. Although some literature suggests that preoperative end-of-life care discussions present a unique chance to start values discussions early and involve loved ones [40], our findings suggest dyads may already be in a supported level of care involvement with the patient having limited decision-making capacity by the time they are faced with decision-making conversations for a medical intervention. This could limit a PWD’s ability to advocate for their values, preferences, and goals. Moreover, our findings highlight the consequences of non-dementia related medical interventions to quality-of-life without ACP for those living with dementia and their carepartners– supporting the literature on ensuring dyads engage in ACP conversions early in disease progression as part of an integrative approach to care, rather than waiting for trigger events such as medical interventions to initiate ACP discussions [41]. ACP timing is often inhibited by the undefined roles of clinicians in the ACP process [42]. However, current research shows much disagreement amongst healthcare providers on who should be initiating ACP conversations and clinical guidelines are unclear [43]. At the same time, preferences for engagement in ACP decision making vary by patient and dyads, some choosing to live day-by-day. Consequently, ACP conversations should be approached as a normalized ongoing process that is revisited by health professionals and concordant with patient values as well as preferences.

The issue of timing is related in addressing the PWD’s decision making capacity, which asks clinicians to understand patients’ capacity to engage and their preferences for carepartner involvement, which are likely to shift as cognitive decline advances [44, 45]. Literature on PWD having to undergo surgery establishes that assessing decision-making capacity is necessary, but clearer guidance on how to make decisions based on PWDs capacity while further considering the carepartners’ values and level of involvement is still warranted [46, 47]. Our findings highlight shifting levels of carepartner involvement in decision-making as participants move from shared to supported to surrogate decision-making. In line with previous research, this study found an increasing amount of carepartner involvement in decision-making as symptoms of dementia progressed, demonstrating an association between mental capacity and participation in medical decision-making [15, 31].

Ours is among the first studies to focus on questions related to medical intervention decision-making among PWD and their carepartners. There is mixed evidence around carepartners’ of PWD accurateness in representing their loved ones in decision-making processes [48]. Our findings align with studies that have found that carepartners that indicate shared values and trusting relationships can often be relied upon to make decisions around medical procedures and surgery for the patient as they accurately represent their values, preferences and respect their wishes as the disease progresses [49]. Our finding can help inform current developments and implementation of enhanced consent processes for PWD on whether to undergo medical procedures, as they do not explicitly consider the role of the carepartner [50]. Still more research is needed on how to specifically approach conversations within each identified category of involvement without compromising patient autonomy.

Our results found that a trusting relationship with the clinician and multidisciplinary team can facilitate good decision-making. Dyads stated they found decision-making discussions to be most successful when they felt their clinicians genuinely cared about their wellbeing and would engage with them on topics besides their diagnosis because it built trust and was conducive to open communication. In line with previous research, we find that open dialogue helped decrease the power imbalance between the clinician and PWD and facilitate good decision-making conversations [51]. There is a growing body of research on interventions that reduce isolation in decision-making [52]. Our research adds to this work by showing that support systems like support groups and social workers can help to empower patients and carepartners by providing anecdotal knowledge and emotional support for PWD to make better decisions concerning their care. Moving forward, medical decision-making guidelines for PWD should promote access to support groups for dyads before, during and after their medical intervention decision-making process. Guidelines should also offer specific recommendations on how to build trust between dyads and clinicians, such as giving space to open conversations and providing information where appropriate on how surgery may impact cognition.

This study has several limitations. There was an imbalance between patient and carepartner participation due to difficulties including patients with cognitive impairment concerns. COVID-19 augmented limitations in recruitment as few new patients were eligible or chose to participate in our study. The sample was mostly comprised of white, non-Hispanic participants from affluent areas. As such, our study lacks the voices of racial or ethnic minoritized groups that represent important disparities in the ADRD population. All patients were men and vast majority of carepartners were women. The three health systems, one from the Mountain region and two from the Northeast, included in this study restrict participants to certain geographical regions. For these reasons, transferability of the findings may be limited. Although our sampling was limited, we enhanced our qualitative research quality criteria by confirming PWDs diagnosis through medical records, brief cognitive testing and investigator triangulation by deliberating as a group. Future studies should try to expand the spectrum of patients and carepartner experiences examined.

In summary, our findings identify the need to improve guidelines on medical decision-making for dyads. Evidence indicates medical interventions may worsen cognitive decline, exacerbate symptoms, lead to longer hospital stays and increased morbidity among PWD [6, 53]. The consequences of medical interventions affect PWD and carepartners’ quality-of-life. For patients with mild-to-moderate dementia, clinicians should screen for dementia before performing medical interventions that may pose a risk to PWD and have decision-making conversations with dyads around risks and benefits. Patients presenting more severe disease progression can rely more on carepartner involvement and patient-carepartner dynamics to better adapt medical decision-making conversations for PWD. Although medical decisions can enable ACP conversations, clinicians with PWD should aim to have those conversations before disease progression and an ongoing process during a dementia trajectory as to not wait for trigger events such as medical interventions. Finally, relationship building, and a multidisciplinary team can enhance trust and empower dyads to make decisions that best fit their life goals and preferences. Through these adaptations, healthcare delivery for people with dementia can be improved to offer patients better outcomes from decision-making conversations.

## Electronic supplementary material

Below is the link to the electronic supplementary material.

Supplementary Material 1

Supplementary Material 2

Supplementary Material 3

Supplementary Material 4

## Data Availability

We are committed to protecting the privacy and confidentiality of our participants. Data from this study is not publicly available for replication purposes. Our qualitative study contains de identified data, but due to the sample size and sensitive nature of the interviews, there is still a possibility they could be identified by the details of their experiences.

## Abbreviations

PWD: Persons with Dementia
ACP: Advance Care Planning
ADRD: Alzheimer’s Disease and Related Dementias
PT: Patient
CP: Carepartner
DNR: Do not resuscitate
