# Supplementary material for: Medical decision-making experiences of persons with dementia and their carepartners: a qualitative study
Source: BMC Palliat Care. 2025 Apr 9;24:99. doi: 10.1186/s12904-025-01710-9 (PMC11983899; doi:10.1186/s12904-025-01710-9)
Supplement: Supplementary file 2 — Supplementary Material 2 [file 12904_2025_1710_MOESM2_ESM.docx]

| Interviewees: |  |
| --- | --- |
| Role |  |
| Interviewer(s): |  |
| Date of Interview: |  |

**For the care partner/patient, they call: 425-436-6200 access code 283347#**

**For recording interview on phone: 425-436-6200 access code 283347#**

**If you are the host, enter * rather than #**

**Host Pin: 434857***

(To start recording, host hits: *9, then press 1.  To stop recording, host hits: *9, then press 1)

# Introduction

- Thank you for speaking with me/us today. I/we are calling to find out if you are a good fit for doing an interview for our study. What I want to do is explain the study to you and then ask you some questions to find out if we should keep going. Sound good? OK, let’s begin.
  - Introduce yourself/others on call

Identification of who is Participating on the Call

Name of care partner: __________________________________________

Name of patient with memory problem: _____________________________

- I/we are researchers affiliated with Brigham and Women’s Hospital. The goal of this study is to learn about patients’ and family care partners’ experiences thinking about and making decisions about surgery in patients with dementia or helping others facing these decisions.
- We are interested in learning more about the conversations that you have had with your doctors about surgery and advance care planning.
- Advance care planning, or ACP, are conversations between patients, and clinicians (physicians, social workers, nurses, and chaplains), sometimes with a family member or friend, about preferences for future medical care if health gets worse. These conversations try to learn about what patients value, their hopes, goals and concerns about the future that could affect the type of medical care they want or don’t want.
- We are very interested to hear your stories about advance care planning, what is important to you and your family when making healthcare decisions, and particularly what is important to you when considering surgery.
- We are particularly interested in what makes these conversations hard to have, and what might make them easier.
- Your stories will be used to help us understand how people with dementia make decisions about surgery and how to better provide care and help patients facing these decisions.

# Consent/Confidentiality Provisions

***[INTERVIEWER: This section is for care partner and patient with dementia interviews (phone or in-person) and any in-person interviews. YOU MUST READ THE CONSENT LANGUAGE AND OBTAIN VERBAL CONSENT BOTH FOR PARTICIPATION AND FOR AUDIO RECORDING]***

Your participation is voluntary and you are free to decide whether or not to participate and to leave at any time. The main risk of participating is loss of confidentiality, but please be assured that all of your responses will be kept confidential-meaning it will not be shared outside of the group with anyone, including your health care team. We will not associate your name or other identifying information with anything you say during the interview. The potential benefit of participating is helping others who face decisions about surgery when they have dementia. Your participation will not affect ongoing or future care, so be as open and honest as you can. We understand how important it is that this information is kept private and confidential.

For interviews with patients with dementia (with or without care partner), proceed to **Screening for Telephone Interviews of Patients with Dementia** on next page (page 4).

Else, for interviews with **care partner only**, proceed to **Interview Recording Introduction** below.

# Interview Recording Introduction

- We would like to audio-record the interview if that is all right with you, so that we can review transcripts later with all the other interviews. We can stop recording at any time for off the record remarks. Do you agree to have this interview be audio-recorded? (**YES/NO**)

**If yes**: Great. Let’s get started. I’ll start the recording.

[FOR THE INTERVIEWER: PRESS *9 TO START RECORDING, NOTE THEY WILL HEAR MUSIC FOR A FEW MOMENTS. YOU WILL HEAR MUSIC AND THEN A RECORDED VOICE SAYING THAT THE CONVERSATION IS BEING RECORDED. **IF NO**: THAT’S FINE. WE WILL TAKE NOTES – AND NOT TAPE THE DISCUSSION. LET’S GET STARTED.]

# Interview recording begins

- Consent: For the record, please state your name and that you consent to be interviewed and [to be audio recorded]

Thank you for agreeing to do this interview. As we discussed, this will take approximately 45 minutes and I will be asking you questions about advance care planning and surgery. If at any point you wish to stop the interview, or have any questions, please let me know. If you are ready, we will begin now.

**Go to page 6 for beginning of demographic questions to begin care partner only interview.**

**Screening for Telephone Interviews with Patients with Dementia**

**[Goal: Assessment of patient capacity to provide informed consent.]**

***For telephone interviews including the patient with dementia, ask the following questions:***

**We would now like to ask some questions to assess your understanding of this consent process. Is that OK?**

- Do you have any questions? (**YES/NO**)
- Do you have to enroll in this study? (Patient understands that participation in the study is completely voluntary) (**CORRECT/INCORRECT**)
- Can you quit the study after you have agreed to participate? (Patient is aware that he/she can discontinue study participation at any time) (**CORRECT/INCORRECT**)
- Can you please describe in your own words what this study is about? (Patient can describe the purpose of the study) (**CORRECT/INCORRECT**)
- What are the main risks of participation in this study? (Patient understands that there are some small risks associated with the study) (**CORRECT/INCORRECT**)
- What are the potential benefits of participation in this study? (Patient understands that he/she will have no direct benefits from the study) (**CORRECT/INCORRECT**)
- Can you describe what your participation in the study will involve? (Prompts: time commitment, study procedures) (**CORRECT/INCORRECT**)
- If you do not participate in the study, will it affect your medical care? (Patient understands that declining participation will not affect medical care) (**CORRECT/INCORRECT**)
- Will the information you give us for this study be kept secret and confidential within the study personnel and authorized staff? (**CORRECT/INCORRECT**)

Capacity Score: _____________

Patient eligible to answer questions? (score of 8 or above) (**YES/NO**)

**[ ] If patient scores <8 (fail), say “Based on your responses, we think the study is not a good fit for you. Thank you for your time and for speaking with me today.” Can proceed with care partner interview (if applicable).**

[If they ask why? We are looking for patients with different kind of memory issues.]

**[ ] If scores 8/8 (pass), proceed with Interview Recording Instruction on next page (page 5).**

Date of telephone screen consent: _________________________

# Interview Recording Introduction

- We would like to audio-record the interview if that is all right with you, so that we can review transcripts later with all the other interviews. We can stop recording at any time for off the record remarks. Do you agree to have this interview be audio-recorded? (**YES/NO**)

**If yes**: Great. Let’s get started. I’ll start the recording.

[FOR THE INTERVIEWER: PRESS *9 TO START RECORDING, NOTE THEY WILL HEAR MUSIC FOR A FEW MOMENTS. YOU WILL HEAR MUSIC AND THEN A RECORDED VOICE SAYING THAT THE CONVERSATION IS BEING RECORDED. **IF NO**: THAT’S FINE. WE WILL TAKE NOTES – AND NOT TAPE THE DISCUSSION. LET’S GET STARTED.]

# Interview recording begins

- Consent: For the record, please state your name and that you consent to be interviewed and [to be audio recorded]

Thank you for agreeing to do this interview. As we discussed, this will take approximately 45 minutes and I will be asking you questions about advance care planning and surgery. If at any point you wish to stop the interview, or have any questions, please let me know. If you are ready, we will begin now.

# PATIENT (AND CARE PARTNER) DEMOGRAPHIC QUESTIONS

What is your age? ______________ [Care partner: What is your age?]

What is your gender? Male Female Other [Care partner: What is your gender?]

What best describes your race? [Care partner: What best describers your race?]

(Please indicate all that apply)

White

Black or African-American

Asian

Native Hawaiian or Pacific Islander

American Indian or Alaska Native

Other - How would you describe your race? ____________________

Don’t know

Refused

Are you of Hispanic or Latino origin or descent? [Care partner: Are you of Hispanic or Latino origin or descent?]

Yes

No

Don’t know

Refused

What is your grade or year of school you have completed?

[Care partner: What is your grade or year of school you have completed?]

Some high school, but did not graduate

High school graduate or GED

Some college

Two- or four-year degree

Graduate degree

Patient: Who primarily helps you at home and attends your medical appointments?

[Care Partner: Who primarily helps <<pt name>> at home and attends to their medical appointments?]

Spouse

 Son or daughter

 Grandson or granddaughter

 Sibling

 Niece or nephew

 Legal guardian

Other ­­­­­­­­­­­­­­­­­­­­­­­­­­­­­­­­­_______________________________

Patient: Do you have a health care proxy, or someone who would make medical decisions for your care if you are unable to?

[Care Partner: Does <<pt name>> have a health care proxy, or someone who would make medical decisions for your care if they are unable to?]

Yes

No

Don’t know

Refused

Patient: Who is your health care proxy?

[Care partner: Who is <<pt name>>’s health care proxy?>>]

 Spouse

 Son or daughter

 Grandson or granddaughter

 Sibling

 Niece or nephew

 Legal guardian

 None chosen or formally designated

 Other _______________________________

Care partner’s relationship to the patient:

Spouse

 Son or daughter

 Grandson or granddaughter

 Sibling

 Niece or nephew

 Legal guardian

Other ­­­­­­­­­­­­­­­­­­­­­­­­­­­­­­­­­_______________________________

## [Goal: Anchor for Patient and Family Caregiver Current Health Concerns/Priorities]

1. PT/CAREGIVER: Can you help us better understand what your current health concerns and challenges are that you hope to get addressed with medical care?

## [Goal: Satisfaction with Decision-Making Process]

1. PT: What do you consider your most important values, goals, and preferences when you make decisions about your healthcare?

[CARE PARTNER: What do you consider your most important values, goals, and preferences when you make decisions about <<pt name>> healthcare?]

1. PT: Do you feel that your doctors/healthcare providers understand these values, goals and preferences? Does your family/HCP understand?

[CARE PARTNER: Do you feel that your doctors/healthcare providers understand these values, goals and preferences? Does <pt name> understand?]

Probe: Can you tell us about any discussions you may have had with your family care partners/health care proxy (CARE PARTNER: <<pt name>>) while making your health care decisions? How much help or guidance do you seek form them?

1. PT: Could you please describe any experiences you have had with advance care planning? (Probes: How did it come about and what happened as a result? Who was involved in the discussion?)

[CARE PARTNER: Could you please describe any experiences you have had with advance care planning for <<pt name>>? (Probes: How did it come about and what happened as a result? Who was involved in the discussion?)]

1. PT: What types of decisions did you make during your ACP conversations? (Probes: CPR? deferral of decisions to family? Artificial feeding tubes?)

[CARE PARTNER: What types of decisions did you make during your ACP conversations about <<pt name’s> care? (Probes: CPR? deferral of decisions to family? Artificial feeding tubes?)]

1. PT/ CARE PARTNER: Can you tell us about how you arrived at your decisions?
2. PT/ CARE PARTNER: When do you think is the best time to start ACP discussions (i.e. at what point in the dementia journey)? How often should it be re-discussed? At any particular time points/events – why?

Probe: Should advance care planning conversations be routinely discussed before or after surgery?

**PT: We are interested in understanding your experience with taking to your clinician about and making decisions about your recent/upcoming surgery.**

[CARE PARTNER: We are interested in understanding your experience with taking to <pt name> clinician about and making decisions about <pt name> recent/upcoming surgery.]

1. Can you tell us what type of surgery you were/are deciding about? ___________________________________

If surgery in past:

Is there anything that you wish you had known before surgery?

Is there anything that surprised you, that you wish you had known?

Any advice you would give to someone in a similar situation?

What do you think you should ask your doctor about before having surgery?

1. We all have conditions that might make surgery more or less successful – what would you want to know from your doctor about that. Some conditions confer additional risk to surgery, such has heart problems or kidney failure. In some cases, if memory problems are severe enough, this might also affect outcomes from surgery.

- What are the best ways of taking about additional risk associated with these conditions.
- Thinking about your situation, what do you think it is important for patients to know.
- PROBE: Sometimes interventions to address one thing might make other things worse. What would you want to know about this?

## [Goal: Wrap up]

We have come to the end of the interview. Thank you very much for your participation. Is there anything we have not discussed that you would like to add?

ADDITIONAL OLDER QUESTIONS:

## [Goal: Comprehension of Current Disease State? (Based on Nunes et al 2016 Comprehension and Cognitive Load]

1. PT/ CARE PARTNER: Has anyone ever talked to you about a diagnosis of dementia or memory problems?

- If yes, ask “How well do you feel you understand your dementia/memory problems? (If patient/care partner indicates a lack of understanding: What is hard for you to understand? What do you find confusing? What do you want to know or learn more about?)”
- If no, ask “Do you think you have memory problems that need attention from a doctor?”

## Now we’re going to switch gears and talk about values, goals, and advance care planning.

1. PT/ CARE PARTNER: Do you think that your goals and preferences about treatment and care might change as time goes on – why/how? (Sellars et al 2018) <Stability Question>
2. PT: How much flexibility should your family/HCP have if you are in a position where you cannot make decisions for yourself but they are in a position to decide about something you have not specifically discussed/considered? <Leeway question>

[CARE PARTNER: How much flexibility should you have if <<PT NAME>> is in a position where he/she cannot make decisions for him/herself but you are in a position to decide about something you have not specifically discussed with <<pt name>>/considered? <Leeway question>]

PT Probe: If you are unable to make medical decisions for yourself, would it be permissible if your family/HCP made decisions for you and that those decisions are different than your previously expressed preferences and values? Why? <Decisional conflict question>

[CARE PARTNER Probe: If <<pt name>> is unable to make medical decisions for him/herself, would it be permissible if you to make decisions for him/her and that those decisions are different than what his/her previously expressed preferences and values? Why? <Decisional conflict question>]

1. *PT: How would you feel* if you were forced to reverse some medical decision you had previously made - for example a decision about not having your heart restarted (CPR) if it stopped – as a requirement for being allowed to have surgery.

*[*CARE PARTNER*: How would you feel* if you were forced to reverse some medical decision you and <<pt name>> had previously made - for example a decision about not having your heart restarted (CPR) if it stopped – as a requirement for <<pt name>> being allowed to have surgery.***]***
