# Supplementary material for: Medical decision-making experiences of persons with dementia and their carepartners: a qualitative study
Source: BMC Palliat Care. 2025 Apr 9;24:99. doi: 10.1186/s12904-025-01710-9 (PMC11983899; doi:10.1186/s12904-025-01710-9)
Supplement: Supplementary file 4 — Supplementary Material 4 [file 12904_2025_1710_MOESM4_ESM.pdf]

Recruitment and contact

Study ID

____

Caregiver name (First Last)

____

Caregiver phone number

____

caregiver Street, City, State, ZIP

Patient name (First Last)

____

patient phone number

____

patient address

Patient still alive?
- ☐ Yes
- ☐ No

Is patient eligible for interview?
- ☐ Yes
- ☐ No

Why not? __________________________________

Date letter sent __________________________________

Returned due to bad address?
- ☐ Yes
- ☐ No

Able to contact by phone?
- ☐ Yes
- ☐ No

caregiver interested in study?
- ☐ Yes
- ☐ No

reason not interested __________________________________

patient initially eligble for interview?
- ☐ Yes
- ☐ No

Now, we would like to make sure you understand the informed consent document we have sent to you. This is a part of our standard procedure.

Do you have to enroll in this study? (Patient understands that participation in the study is completely voluntary)
- ☐ correct
- ☐ incorrect

Can you quit the study after you have agreed to participate? (Patient is aware that he/she can discontinue study participation at any time)
- ☐ correct
- ☐ incorrect

3. Can you please describe in your own words what this study is about? (Patient can describe the purpose of the study)
- ☐ correct
- ☐ incorrect

4. What are the main risks of participation in this study? (Patient understands that there are some small risks associated with the study)

☐ correct
☐ incorrect

What are the potential benefits of participation in this study? (Patient understands that he/she will have no direct benefits from the study)

☐ correct
☐ incorrect

Can you describe what your participation in the study will involve? [Prompts: time commitment, study procedures]

☐ correct
☐ incorrect

If you do not participate in the study, will it affect your medical care? Patient understands that declining participation will not affect medical care.

☐ correct
☐ incorrect

Will the information you give us for this study be kept secret and confidential within the study personnel and authorized staff?

☐ correct
☐ incorrect

capacity score

____

Patient eliblge to answer questions? (score of 6 or above)

☐ Yes
☐ No

Date of telephone consent

____

Caregiver Information

Interview Date

____

Thank you for agreeing to do this interview. As we discussed, this will take approximately 45 minutes and I will be asking you questions about your role as a caregiver to someone with dementia. If at any point you wish to stop the interview, or have any questions, please let me know. If you are ready we will begin now. Before I ask you any questions, can you please tell me the first name of the person you care for so I can refer to him or her.

Now I'd like to ask a few questions about you.

What is your age?

____

What is your gender?

☐ Male ☐ Female

What best describes your race?

☐ American Indian/Alaska native
☐ Asian
☐ Native Hawaiian or other Pacific Islander
☐ Black/African American
☐ White
☐ Other
☐ Don't know
☐ Refused

How would you describe your race?

____

What best describes you ethnicity?

☐ Hispanic/Latino
☐ Not Hispanic/Latino
☐ Don't know
☐ Refused

What is the grade or year of school you have completed?

☐ No schooling
☐ Not past 8th grade
☐ 9-11 grade
☐ High school
☐ Technical or trade school
☐ Some college
☐ Bachelor's degree
☐ Graduate degree
☐ Refused

What is your relationship to [patient]?

☐ Spouse
☐ Son or daughter
☐ Grandson or granddaughter
☐ Sibling
☐ Niece or nephew
☐ Legal guardian
☐ Other

____

(Specify 'other' relationship)

How often do you provide care, or help with day to day tasks such as transportation, shopping, using the phone, housekeeping or preparing meals for [patient]?

☐ Every day
☐ At least once a week but not every day
☐ Occasionally but not every week
☐ Not at all
☐ Refused

How long have you provided care for [patient]?

☐ Less than 6 months
☐ Between six months and one year
☐ Between one year and 3 years
☐ More than 3 years
☐ Other

____
(How long have you been a caregiver for [patient]?)

Goals Of Care

As you know, [patient] has been diagnosed with dementia, which is a progressive illness. As the disease progresses, there will likely come a time when [patient] is no longer able to participate in decisions regarding [his/her] medical care. As someone who has already been involved in medical decisions for [patient], we would like to ask you some questions about any communication or counseling you have had to help you make these decisions.

OVERALL GOALS OF CARE

First, I would like to ask you about [patient's] overall goals for [his/her] medical care.

If [patient] had to make a choice now, do you think that [he/she] would prefer future treatments that extend life, even if that means having more pain or discomfort, or would [he/she] prefer treatments that focus on relieving pain and discomfort even if it means not living as long?

☐ Extend life ☐ Relieve pain or discomfort ☐ Don't know ☐ Refused

Has [patient] ever expressed to you [his/her] wishes regarding this sort of decision?

☐ Yes ☐ No ☐ Don't know ☐ Refused

When?

☐ In the last month
☐ In the last 6 months
☐ In the last year
☐ More than one year ago
☐ Other

When?

____

As far as you know, has a doctor ever discussed these preferences for care with [patient]?

☐ Yes ☐ No ☐ Don't know ☐ Refused

When?

☐ In the last month
☐ In the last 6 months
☐ In the last year
☐ More than one year ago
☐ Other

____
(When?)

Do you think there should be a future discussion with [his/her] doctor about preferences for care?

☐ Yes ☐ No ☐ Don't know ☐ Refused

SPECIFIC TREATMENTS

There are a number of treatments that can be given to patients. I am going to describe several treatments to you, and for each one I will ask you whether [patient] has told you whether [he/she] would want this treatment in the future. I am also going to ask whether a doctor has explained this treatment and discussed with [patient] or you whether [patient] would want this treatment.

Resuscitation

Resuscitation means trying to restart the heart and using a machine to help breathing. Has [patient] expressed a preference regarding resuscitation?

☐ Yes ☐ No ☐ Don't Know ☐ Refused

What is that preference?

☐ Wants resuscitation under any circumstance ☐ Does not want resuscitation under any circumstance
☐ Depends on circumstance ☐ Don't know ☐ Refused

When was this preference expressed?

☐ In the last month
☐ In the last 6 months
☐ In the last year
☐ More than one year ago
☐ Other

____
(When?)

Has a doctor ever explained resuscitation and discussed [patient's] preferences with [patient]?

☐ Yes ☐ No ☐ Don't know ☐ Refused

Do you think there should be a future discussion with [his/her] doctor regarding resuscitation?

☐ Yes ☐ No ☐ Don't know ☐ Refused

Intubation

Intubation means placing a breathing tube into the lungs. Has [patient] expressed a preference regarding intubation?

☐ Yes
☐ No
☐ Don't Know
☐ Refused

What is that preference?

☐ Wants to be intubated under any circumstance ☐ Does not want to be intubated under any circumstance
☐ Depends on circumstance ☐ Don't know ☐ Refused

When was this preference expressed?

☐ In the last month
☐ In the last 6 months
☐ In the last year
☐ More than one year ago
☐ Other

____
(When?)

Has a doctor ever explained intubation and discussed [patient's] preferences with [patient]?

☐ Yes ☐ No ☐ Don't know ☐ Refused

Do you think there should be a future discussion with [his/her] doctor regarding intubation?

☐ Yes ☐ No ☐ Don't know ☐ Refused

Hospitalization

Has [patient] expressed any preference regarding staying overnight in the hospital?

☐ Yes ☐ No ☐ Don't Know ☐ Refused

What is that preference?

☐ Always wants to be hospitalized ☐ Never wants to be hospitalization ☐ Depends on circumstances
☐ Don't know ☐ Refused

When was this preference expressed?

☐ In the last month
☐ In the last 6 months
☐ In the last year
☐ More than one year ago
☐ Other

____
(When?)

Has a doctor discussed [patient's] preferences regarding hospitalization with [patient]?

☐ Yes ☐ No ☐ Don't know ☐ Refused

Do you think there should be a future discussion with [his/her] doctor regarding hospitalization?

☐ Yes ☐ No ☐ Don't know ☐ Refused

Tube Feeding

Tube feeding involves placing a tube into the stomach through which liquid food is given. Has [patient] expressed a preference regarding tube feeding?

☐ Yes ☐ No ☐ Don't Know ☐ Refused

What is that preference?

☐ Wants tube feeding under any circumstance
☐ Does not want tube feeding under any circumstance
☐ Depends on circumstances
☐ Don't know
☐ Refused

When was this preference expressed?

☐ In the last month
☐ In the last 6 months
☐ In the last year
☐ More than one year ago
☐ Other

____
(When?)

Has a doctor ever explained tube feeding and discussed [patient's] preferences with [patient]?

☐ Yes ☐ No ☐ Don't know ☐ Refused

Do you think there should be a future discussion with [his/her] doctor regarding tube feeding?

☐ Yes ☐ No ☐ Don't know ☐ Refused

Hospice

Hospice is a type of care focused on comfort for persons of limited life expectancy. Has [patient] expressed a preference regarding hospice?

☐ Yes ☐ No ☐ Don't Know ☐ Refused

What is that preference?

☐ Wants hospice ☐ Does not want hospice ☐ Don't know ☐ Refused

When was this preference expressed?

☐ In the last month
☐ In the last 6 months
☐ In the last year
☐ More than one year ago
☐ Other

____
(When?)

Has a doctor ever explained hospice and discussed [patient's] preferences with [patient]?

☐ Yes ☐ No ☐ Don't know ☐ Refused

Do you think there should be a future discussion with [his/her] doctor regarding hospice?

☐ Yes ☐ No ☐ Don't know ☐ Refused

Understanding End Stage Dementia

I would like to ask you some questions about dementia, and how the illness affects people over time.

In your own words, can you tell me what you know about what happens to patients at the end stages of dementia?

How well do you feel you understand what happens to patients at the end stage of illness?

☐ Very well
☐ Somewhat well
☐ Not very well
☐ Not at all well

How interested are you in learning more about what happens at this time?

☐ Very interested
☐ Somewhat interested
☐ Not very interested
☐ Not at all interested

When do you think would be the best time to tell patients and families about what to expect at the end stage of dementia?

☐ When they are first told of the diagnosis
☐ After they have been aware of having the disease for a few years
☐ When they start to enter the end stage of the disease
☐ Other

(When should they be told what to expect)

To what degree do you feel [patient] is able to participate in decisions regarding his/her health care?	☐ not at all able<br>☐ somewhat able<br>☐ fully able<br>☐ don't know<br>☐ refused
To what degree do you feel [patient] will be able to participate in decisions regarding his/her health care one year from now?	☐ not at all able<br>☐ somewhat able<br>☐ fully able<br>☐ don't know<br>☐ refused

Proxy Living Will

HEALTHCARE PROXY

When patients know they may not be able to make medical decisions in the future, they sometimes designate someone else who will make those decisions. This person is often called a health care proxy. In order to designate a health care proxy, a person has to fill out and sign some paperwork.

As far as you know, has (patient) completed any paperwork to designate a health care proxy?

☐ Yes ☐ No ☐ Don't know ☐ Refused

When was this paperwork completed?

☐ In the last month
☐ In the last 6 months
☐ In the last year
☐ More than one year ago
☐ Other

____
(When?)

Are you the proxy?

☐ Yes ☐ No ☐ Don't know ☐ Refused

The following questions ask about how confident you are to actually talk to [patient] about their medical wishes. The 5 possible answers are "not at all, a little, somewhat, fairly, extremely

How confident are you that today you could agree to be [patient's] medical decision maker?

☐ Not at all
☐ A little
☐ somewhat
☐ fairly
☐ extremely
☐ not sure

How confident are you that today you could talk with [patient] about the kind of medical care he/she would want if they were seriously ill or dying?

☐ Not at all
☐ a little
☐ somewhat
☐ fairly
☐ extremely
☐ not sure

How confident are you that today you could talk with [patient] about how much flexibility they would want you to have in making medical decisions for them?

☐ Not at all
☐ a little
☐ somewhat
☐ fairly
☐ extremely
☐ not sure

How confident are you that today you could talk with [patient's] doctors about the kind of medical care he/she would want if they were seriously ill or dying?

☐ Not at all
☐ a little
☐ somewhat
☐ fairly
☐ extremely
☐ not sure

How confident are you that today you could ask the right questions of [patient's] doctors to help make good medical decisions?

- ☐ Not at all
- ☐ a little
- ☐ somewhat
- ☐ fairly
- ☐ extremely
- ☐ not sure

The following questions ask about how ready you are to do something. The options are: 1) I have never thought about it; 2) I have thought about it, but I am not ready to do it; 3) I am thinking about doing it in the next 6 months; 4) I am planning to do it in the next 30 days; 5) I already did it

How ready are you to formally agree to be [patient's] medical decision maker?

- ☐ I have never thought about it
- ☐ I have thought about it, but I am not ready to do it
- ☐ I am thinking about doing it in the next 6 months
- ☐ I am planning to do it in the next 30 days
- ☐ I already did it

How ready are you to have [patient] sign official papers naming you as the person to make medical decisions for them?

- ☐ I have never thought about it
- ☐ I have thought about it, but I am not ready to do it
- ☐ I am thinking about doing it in the next 6 months
- ☐ I am planning to do it in the next 30 days
- ☐ I already did it

How ready are you to talk to with [patient] about how much flexibility they want to give you in making medical decisions for them?

- ☐ I have never thought about it
- ☐ I have thought about it, but I am not ready to do it
- ☐ I am thinking about doing it in the next 6 months
- ☐ I am planning to do it in the next 30 days
- ☐ I already did it

How ready are you to talk to [patient] about the kind of medical care they would want if they were seriously ill or dying?

- ☐ I have never thought about it
- ☐ I have thought about it, but I am not ready to do it
- ☐ I am thinking about doing it in the next 6 months
- ☐ I am planning to do it in the next 30 days
- ☐ I already did it

How ready are you to talk to [patient's] doctor about the kind of medical care they would want if they were seriously ill or dying?

- ☐ I have never thought about it
- ☐ I have thought about it, but I am not ready to do it
- ☐ I am thinking about doing it in the next 6 months
- ☐ I am planning to do it in the next 30 days
- ☐ I already did it

LIVING WILL

Sometimes, patients fill out paperwork to say what sort of medical treatments they do or do not want in the future. This type of document is often called a living will.

As far as you know, has [patient] completed a written living will or similar document?

☐ Yes ☐ No ☐ Don't know ☐ Refused

When was this paperwork completed?

- ☐ In the last month
- ☐ In the last 6 months
- ☐ In the last year
- ☐ More than one year ago
- ☐ Other

____
(When?)

Medical History And Decisions

Now I am going to ask you some questions about [patient's] medical history and your involvement in related decisions.

Have you been involved in helping to make any medical decisions for [patient] in the last six months?

☐ Yes
☐ No
☐ Don't know
☐ Refused

Can you describe the last decision you remember being involved in? ______________________________

Over the past 6 months, as far as you know, has [patient] gone to the hospital or emergency room?

☐ Yes ☐ No ☐ Don't know ☐ Refused

Were you involved in that medical decision?

☐ Yes ☐ No ☐ Don't know ☐ Refused

Over the past 6 months, as far as you know, has [patient] been resuscitated or intubated?

☐ Yes ☐ No ☐ Don't know ☐ Refused

Were you involved in that medical decision?

☐ Yes ☐ No ☐ Don't know ☐ Refused

Over the past 6 months, as far as you know, has [patient] been a candidate for surgery?

☐ Yes ☐ No ☐ Don't know ☐ Refused

Were you involved in that medical decision?

☐ Yes ☐ No ☐ Don't know ☐ Refused

Understanding Dementia

Now I am going to read some statements about Alzheimer's disease or dementia. Please answer whether you think the statement is true or false

People with Alzheimer's disease are particularly prone to depression.

☐ True ☐ False

It has been scientifically proven that mental exercise can prevent a person from getting Alzheimer's disease.

☐ True ☐ False

After symptoms of Alzheimer's disease appear, the average life expectancy is 6 to 12 years.

☐ True ☐ False

When a person with Alzheimer's disease becomes agitated, a medical examination might reveal other health problems that caused the agitation.

☐ True ☐ False

People with Alzheimer's disease do best with simple instructions given one step at a time.

☐ True ☐ False

When people with Alzheimer's disease begin to have difficulty taking care of themselves, caregivers should take over right away.

☐ True ☐ False

If a person with Alzheimer's disease becomes alert and agitated at night, a good strategy is to try to make sure that the person gets plenty of physical activity during the day.

☐ True ☐ False

In rare cases, people have recovered from Alzheimer's disease.

☐ True ☐ False

People whose Alzheimer's disease is not yet severe can benefit from psychotherapy for depression and anxiety.

☐ True ☐ False

If trouble with memory and confused thinking appears suddenly, it is likely due to Alzheimer's disease.

☐ True ☐ False

Most people with Alzheimer's disease live in nursing homes.

☐ True ☐ False

Poor nutrition can make the symptoms of Alzheimer's disease worse.

☐ True ☐ False

People in their 30's can have Alzheimer's disease.

☐ True ☐ False

A person with Alzheimer's disease becomes increasingly likely to fall down as the disease gets worse.

☐ True ☐ False

When people with Alzheimer's disease repeat the same question or story several times, it is helpful to remind them that they are repeating themselves.

☐ True ☐ False

Once people have Alzheimer's disease, they are no longer capable of making informed decisions about their own care.

☐ True ☐ False

Eventually, a person with Alzheimer's disease will need 24-hour supervision.

☐ True ☐ False

Having high cholesterol may increase a person's risk of developing Alzheimer's disease.

☐ True ☐ False

Tremor or shaking of the hands or arms is a common symptom in people with Alzheimer's disease.

☐ True ☐ False

Symptoms of severe depression can be mistaken for symptoms of Alzheimer's disease.

☐ True ☐ False

Alzheimer's disease is one type of dementia.

☐ True ☐ False

Trouble handling money or paying bills is a common early symptom of Alzheimer's disease.

☐ True ☐ False

One symptom that can occur with Alzheimer's disease is believing that other people are stealing one's things.

☐ True ☐ False

When a person has Alzheimer's disease, using reminder notes is a crutch that can contribute to decline.

☐ True ☐ False

Prescription drugs that prevent Alzheimer's disease are available.

☐ True ☐ False

Having high blood pressure may increase a person's risk of developing Alzheimer's disease.

☐ True ☐ False

Genes can only partially account for the development of Alzheimer's disease.

☐ True ☐ False

It is safe for people with Alzheimer's disease to drive, as long as they have a companion in the car at all times.

☐ True ☐ False

Alzheimer's disease cannot be cured.

☐ True ☐ False

Most people with Alzheimer's disease remember recent events better than things that happened in the past.

☐ True ☐ False

Acceptance Of Illness

I am going to ask you some questions about how you feel about [patient's] illness. The answer choices are part of a four-point scale ranging from "Not at all" to "To a large extent". "To a slight extent" and "To some extent" are in the middle.

To what extent are you able to accept [patient]'s diagnosis of dementia?

☐ Not at all
☐ To a slight extent
☐ To some extent
☐ To a large extent

To what extent would you say you have a sense of inner peace and harmony?

☐ Not at all
☐ To a slight extent
☐ To some extent
☐ To a large extent

To what extent do you feel that you have made peace with [patient]'s illness?

☐ Not at all
☐ To a slight extent
☐ To some extent
☐ To a large extent

Do you feel well loved now?

☐ Not at all
☐ To a slight extent
☐ To some extent
☐ To a large extent

To what extent do you feel a sense of inner calm and tranquility?

☐ Not at all
☐ To a slight extent
☐ To some extent
☐ To a large extent

Struggle with Illness Subscale

To what extent do changes in [patient]'s physical appearance upset you?

☐ Not at all
☐ To a slight extent
☐ To some extent
☐ To a large extent

To what extent does worry about [patient]'s illness make it difficult for you to live from day to day?

☐ Not at all
☐ To a slight extent
☐ To some extent
☐ To a large extent

To what extent do you feel that it is unfair for [patient] to get dementia now?

☐ Not at all
☐ To a slight extent
☐ To some extent
☐ To a large extent

To what extent do you feel that [patient]'s life, as you know it, is now over?

☐ Not at all
☐ To a slight extent
☐ To some extent
☐ To a large extent

To what extent do you feel angry because of [patient]'s illness?

☐ Not at all
☐ To a slight extent
☐ To some extent
☐ To a large extent

To what extent do you think [patient]'s illness has beaten you down?

☐ Not at all
☐ To a slight extent
☐ To some extent
☐ To a large extent

To what extent do you feel ashamed of, or embarrassed by, [patient]'s current condition?

☐ Not at all
☐ To a slight extent
☐ To some extent
☐ To a large extent

Preparedness For Caregiving

I am going to ask you some questions about how prepared you feel you are for being a caregiver for [patient]. The answer choices come from a 5-point scale ranging from "Not at all prepared" to "Very well prepared" with "Not too well prepared", "Somewhat well prepared" and "Pretty well prepared" in between.

How well prepared do you think you are to take care of [patient]s' physical needs

☐ Not at all prepared
☐ Not too well prepared
☐ Somewhat well prepared
☐ Pretty well prepared
☐ Very well prepared

How well prepared do you think you are to take care of [his/her] emotional needs?

☐ Not at all prepared
☐ Not too well prepared
☐ Somewhat well prepared
☐ Pretty well prepared
☐ Very well prepared

How well prepared do you think you are to find out about and set up services for [him/her]?

☐ Not at all prepared
☐ Not too well prepared
☐ Somewhat well prepared
☐ Pretty well prepared
☐ Very well prepared

How well prepared do you think you are for the stress of caregiving?

☐ Not at all prepared
☐ Not too well prepared
☐ Somewhat well prepared
☐ Pretty well prepared
☐ Very well prepared

How well prepared do you think you are to make caregiving activities pleasant for both you and [patient]?

☐ Not at all prepared
☐ Not too well prepared
☐ Somewhat well prepared
☐ Pretty well prepared
☐ Very well prepared

How well prepared do you think you are to respond to and handle emergencies that involve [him/ her]?

☐ Not at all prepared
☐ Not too well prepared
☐ Somewhat well prepared
☐ Pretty well prepared
☐ Very well prepared

How well prepared do you think you are to get the help and information you need from the health care system?

☐ Not at all prepared
☐ Not too well prepared
☐ Somewhat well prepared
☐ Pretty well prepared
☐ Very well prepared

Overall, how well prepared do you think you are to care for [patient]?

☐ Not at all prepared
☐ Not too well prepared
☐ Somewhat well prepared
☐ Pretty well prepared
☐ Very well prepared

Caregiver Burden

I am going to ask you some questions about what it is like to take care of [patient]. The answer choices will be from a 5- point scale ranging from "Never" to "Nearly always", with "Rarely", "Sometimes", and "Quite frequently" in between.

How often do you feel that because of the time you spend with [patient] that you don't have enough time for yourself?

☐ Never
☐ Rarely
☐ Sometimes
☐ Quite frequently
☐ Nearly always

How often do you feel stressed between caring for [patient] and trying to meet other responsibilities (work/family)?

☐ Never
☐ Rarely
☐ Sometimes
☐ Quite frequently
☐ Nearly always

How often do you feel angry when you are around [patient]?

☐ Never
☐ Rarely
☐ Sometimes
☐ Quite frequently
☐ Nearly always

How often do you feel that [patient] currently affects your relationship with family members or friends in a negative way?

☐ Never
☐ Rarely
☐ Sometimes
☐ Quite frequently
☐ Nearly always

How often do you feel strained when you are around [patient]?

☐ Never
☐ Rarely
☐ Sometimes
☐ Quite frequently
☐ Nearly always

How often do you feel that your health has suffered because of your involvement with [patient]?

☐ Never
☐ Rarely
☐ Sometimes
☐ Quite frequently
☐ Nearly always

How often do you feel that you don't have as much privacy as you would like because of [patient]?

☐ Never
☐ Rarely
☐ Sometimes
☐ Quite frequently
☐ Nearly always

How often do you feel that your social life has suffered because you are caring for [patient]?

☐ Never
☐ Rarely
☐ Sometimes
☐ Quite frequently
☐ Nearly always

How often do you feel that you have lost control of your life since [patient]'s illness?

☐ Never
☐ Rarely
☐ Sometimes
☐ Quite frequently
☐ Nearly always

How often do you feel uncertain about what to do about [patient]?

☐ Never
☐ Rarely
☐ Sometimes
☐ Quite frequently
☐ Nearly always

How often do you feel you should be doing more for [patient]?

☐ Never
☐ Rarely
☐ Sometimes
☐ Quite frequently
☐ Nearly always

How often do you feel you could do a better job in caring for [patient]?

☐ Never
☐ Rarely
☐ Sometimes
☐ Quite frequently
☐ Nearly always

Decision Self Efficacy

I would like to ask you about how confident you feel in participating in medical decisions for [patient]. I am going to read some statements about making an informed choice. Please indicate how confident you feel in doing these things by selecting a number from 0 to 4 - with 0 being not at all confident and 4 being very confident.

I feel confident that I can get the facts about the medical choices available to [PATIENT].
(Not at all confident 0 - 4 Very confident)

☐ 0 ☐ 1 ☐ 2 ☐ 3 ☐ 4

I feel confident that I can get the facts about the benefits of each choice.
(Not at all confident 0 - 4 Very confident)

☐ 0 ☐ 1 ☐ 2 ☐ 3 ☐ 4

I feel confident that I can get the facts about the risks and side effects of each choice.
(Not at all confident 0 - 4 Very confident)

☐ 0 ☐ 1 ☐ 2 ☐ 3 ☐ 4

I feel confident that I can understand the information enough to be able to make a choice.
(Not at all confident 0 - 4 Very confident)

☐ 0 ☐ 1 ☐ 2 ☐ 3 ☐ 4

I feel confident that I can ask questions without feeling dumb.
(Not at all confident 0 - 4 Very confident)

☐ 0 ☐ 1 ☐ 2 ☐ 3 ☐ 4

I feel confident that I can express my concerns about each choice.
(Not at all confident 0 - 4 Very confident)

☐ 0 ☐ 1 ☐ 2 ☐ 3 ☐ 4

I feel confident that I can ask for advice.
(Not at all confident 0 - 4 Very confident)

☐ 0 ☐ 1 ☐ 2 ☐ 3 ☐ 4

I feel confident that I can figure out the choice that best suits [patient].
(Not at all confident 0 - 4 Very confident)

☐ 0 ☐ 1 ☐ 2 ☐ 3 ☐ 4

I feel confident that I can handle unwanted pressure from others in making my choice.
(Not at all confident 0 - 4 Very confident)

☐ 0 ☐ 1 ☐ 2 ☐ 3 ☐ 4

I feel confident that I can let the medical team know what is best for [patient].
(Not at all confident 0 - 4 Very confident)

☐ 0 ☐ 1 ☐ 2 ☐ 3 ☐ 4

I feel confident that I can delay my decision if I feel I need more time.
(Not at all confident 0 - 4 Very confident)

☐ 0 ☐ 1 ☐ 2 ☐ 3 ☐ 4

We've now reached the end of the questions. Thank you for your participation. (If patient to be interviewed), I am now ready to speak briefly with (name). ____________________________

Patient Data

Age

____

Race

☐ American Indian/Alaska native ☐ Asian ☐ Native Hawaiian or other Pacific Islander
☐ Black/African American ☐ White ☐ Other ☐ Don't know

Ethnicity

☐ Hispanic/Latino ☐ Not Hispanic/Latino ☐ Don't know

Gender

☐ Male ☐ Female

Grade or year of school completed

☐ No schooling ☐ Not past 8th grade ☐ 9-11 grade ☐ High school ☐ Technical or trade school
☐ Some college ☐ Bachelor's degree ☐ Graduate degree

Dementia severity measure and score __________________________________

Year of dementia test __________________________________

What is patient's site of care?

☐ NewBridge on the Charles
☐ Center communities of Brookline
☐ Orchard Cove
☐ other

Who is patient's provider?

☐ Brinkerhoff
☐ Kandel
☐ Rhoads-kropf
☐ Imanishi
☐ other

other provider __________________________________

Patient goals of care

I would like to ask you some questions about your goals for future health care. If you had to make a choice now, do you think that you would prefer future treatment to extend life, even if that means having more pain or discomfort, or would you prefer treatments that focus on relieving pain and discomfort even if it means not living as long?

☐ Extend life ☐ Relieve pain or discomfort ☐ Don't know ☐ Refused

Have you ever expressed your wishes regarding this sort of decision to a family member or other caregiver?

☐ Yes ☐ No ☐ Don't know ☐ Refused

When?

☐ In the last month
☐ In the last 6 months
☐ In the last year
☐ More than one year ago
☐ Other

Have you ever discussed your wishes regarding this sort of decision with your doctor?

☐ Yes ☐ No ☐ Don't know ☐ Refused

When?

☐ In the last month
☐ In the last 6 months
☐ In the last year
☐ More than one year ago
☐ Other

I would like to ask you some questions about dementia, and how the illness affects people over time.

In your own words, can you tell me what you know about what happens to patients at the end stages of dementia?

How well do you feel you understand what happens to patients at the end stage of illness?

☐ Very well
☐ Somewhat well
☐ Not very well
☐ Not at all well

How interested are you in learning more about what happens at this time?

☐ Very interested
☐ Somewhat interested
☐ Not very interested
☐ Not at all interested

When do you think would be the best time to tell patients and families about what to expect at the end stage of dementia?

☐ When they are first told of the diagnosis
☐ After they have been aware of having the disease for a few years
☐ When they start to enter the end stage of the disease
☐ Other

(When should they be told what to expect)

Confidence and readiness

I would like to ask you some questions having someone help make medical decisions for you in the future. The following questions ask about how confident you are to actually talk to someone about your medical wishes. The 5 possible answers are "not at all, a little, somewhat, fairly, extremely"

How confident are you that today you could ask someone to be your medical decision maker?

- ☐ not at all
- ☐ a little
- ☐ somewhat
- ☐ fairly
- ☐ extremely
- ☐ not sure

How confident are you that today you could talk with your decision maker about the kind of medical care you'd want if you were seriously ill or dying?

- ☐ not at all
- ☐ a little
- ☐ somewhat
- ☐ fairly
- ☐ extremely
- ☐ not sure

How confident are you that today you could talk with your decision maker about how much flexibility you want to give them to make medical decisions for you?

- ☐ not at all
- ☐ a little
- ☐ somewhat
- ☐ fairly
- ☐ extremely
- ☐ not sure

How confident are you that today you could talk with your doctors about the kind of medical care you'd want if you were seriously ill or dying?

- ☐ not at all
- ☐ a little
- ☐ somewhat
- ☐ fairly
- ☐ extremely
- ☐ not sure

How confident are you that today you could sign official papers about the kind of medical care you'd want if you were seriously ill or dying?

- ☐ not at all
- ☐ a little
- ☐ somewhat
- ☐ fairly
- ☐ extremely
- ☐ not sure

How confident are you that today you could ask the right questions of your doctors to help make good medical decisions?

- ☐ not at all
- ☐ a little
- ☐ somewhat
- ☐ fairly
- ☐ extremely
- ☐ not sure

The following questions ask about how ready you are to do something. The options are: 1) I have never thought about it; 2) I have thought about it, but I am not ready to do it; 3) I am thinking about doing it in the next 6 months; 4) I am planning to do it in the next 30 days; 5) I already did it.

How ready are you to formally ask someone to be your medical decision maker?

- ☐ I have never thought about it
- ☐ I have thought about it, but I am not ready to do it
- ☐ I am thinking about doing it in the next 6 months
- ☐ I am planning to do it in the next 30 days
- ☐ I already did it

How ready are you to sign official papers naming a person to make medical decisions for you?

- ☐ I have never thought about it
- ☐ I have thought about it, but I am not ready to do it
- ☐ I am thinking about doing it in the next 6 months
- ☐ I am planning to do it in the next 30 days
- ☐ I already did it

How ready are you to talk to your medical decision maker about how much flexibility you want to give them to make medical decisions for you?

- ☐ I have never thought about it
- ☐ I have thought about it, but I am not ready to do it
- ☐ I am thinking about doing it in the next 6 months
- ☐ I am planning to do it in the next 30 days
- ☐ I already did it

How ready are you to talk to your medical decision maker about the kind of medical care you'd want if you were seriously ill or dying?

- ☐ I have never thought about it
- ☐ I have thought about it, but I am not ready to do it
- ☐ I am thinking about doing it in the next 6 months
- ☐ I am planning to do it in the next 30 days
- ☐ I already did it

How ready are you to talk to your doctor about the kind of medical care you'd want if you were seriously ill or dying?

- ☐ I have never thought about it
- ☐ I have thought about it, but I am not ready to do it
- ☐ I am thinking about doing it in the next 6 months
- ☐ I am planning to do it in the next 30 days
- ☐ I already did it

How ready are you to sign official papers about the kind of medical care you'd want if you were seriously ill or dying?

- ☐ I have never thought about it
- ☐ I have thought about it, but I am not ready to do it
- ☐ I am thinking about doing it in the next 6 months
- ☐ I am planning to do it in the next 30 days
- ☐ I already did it

We've now reached the end of the questions. Thank you for your participation.

____
